# W. George Beers, L.D.S., D.D.S.

**Published:** 1901-02

**Authors:** 


### Obituary.

### W. GEORGE BEERS, L.D.S., D.D.S.

DIED December 26, 1900, Dr. W. George Beers, of Montreal, Canada.

Death has claimed many of the active workers in the dental profession during the past few years, but of these Dr. Beers will not be counted among the least. His ability, activity, and untiring devotion to his profession made his name prominent in Canada and throughout the United States.

He has been connected with the periodical literature of dentistry for many years, and his pungent editorials, well directed against violators of professional ethics, were eagerly read by his colleagues in all sections and extensively quoted. His work, however, was not confined to the *Dominion Dental Journal,* of which he was the principal editor at the time of his death, but extended to an active participation in the general literature of his profession. He was also a contributor to the leading magazines,—*Scribner, Century,* etc. This literary taste was manifested when quite young, having contributed in 1862-63 articles to Wilkes's *Spirit of the Times* on Canadian sports.

Dr. Beers was born in Montreal, May 5, 1843. He was educated at the Lower Canada College and at Phillips School, in that city, and subsequently entered the dental profession. He was the founder of the first dental journal in Canada. He occupied for eleven years the position of Secretary of the Dental Board of

Examiners for the Province of Quebec, and at one time filled the position of Dean of the Provincial Dental College.

It is due mainly to his efforts that dentistry in Canada has assumed its present advanced standard. He was fortunate in having for his colleagues men in thorough sympathy with him in his efforts to elevate dentistry, with the result that the profession there has not only earned the respect of its colaborers elsewhere, but has furnished a standard worthy of emulation.

There is a small class found in all callings who are born leaders, not from selfish motives, but that the work of their choice may advance through their efforts. It always seemed to the writer that Dr. Beers was pre-eminently a leader of this character, strong in his convictions, at times dealing sledge-hammer blows, but always with the one idea that dentistry might be made worthy the respect of men. He sacrificed himself in time, money, and strength to accomplish this, and doubtless died, as such men always die, with little to show in earthly riches.

The writer's personal acquaintance with Dr. Beers dates back many years. His last letter, received some two months since, expressed the same untiring desire for extended work. He thought the time had come when dental periodical literature should take another change, and that from monthly to weekly issues. He had great faith in the success of such a venture. The writer, while failing to agree with him as to the wisdom of such a move, could not help a degree of admiration for his faith in his professional colleagues.

It is presumed the fact is not generally known among Dr. Beers's friends outside of Canada, that he was a devoted athlete. He reduced the game of Lacrosse to a system, and through his efforts it was made a national game in Canada. He organized the first Canadian Lacrosse team to visit England. This was in 1876, commencing to play in Belfast, and from there in the principal towns of Ireland, Scotland, and England. At the command of the Queen, a game was played before her at Windsor Castle. He repeated this visit to England in 1883, and for the same purpose.

He was also active in local military circles, being captain of a company in the Victoria Volunteer Rifles.

His patriotism was of the vigorous sort, and he did not hesitate to give his opinion of those he regarded as violating international comity. At Syracuse, N. Y., in October, 1888, in a public address, he severely castigated the United States for what he considered serious derelictions of duty. This received wide notice and comment.

His patriotism could at times present itself in a more sentimental form. It is related of him that when the Princess Louise and the Marquis of Lorne visited Montreal in 1878, they passed under an arch erected in front of Dr. Beers's house. As the carriage passed under the arch, Dr. Beers pulled a string attached to a basket of flowers, showering the royal couple.

Every good man in all ranks is sadly missed when the veil is drawn down on his life, but few, it is thought, will have passed beyond the borderland more sincerely mourned than the subject of this sketch. He will be sadly missed in this transition period of dentistry, for strong men are needed to carry the profession safely forward into the twentieth century, men who cannot be bought or influenced, but whose aim is always for the highest. Of such was W. George Beers.

His funeral took place at Montreal, December 28, and was largely attended by the various associations with which he was connected.

